# Multidrug-Resistant *Klebsiella pneumoniae* Strains in a Hospital: Phylogenetic Analysis to Investigate Local Epidemiology

**DOI:** 10.3390/microorganisms12122541

**Published:** 2024-12-10

**Authors:** Maria Vittoria Ristori, Fabio Scarpa, Daria Sanna, Marco Casu, Nicola Petrosillo, Umile Giuseppe Longo, De Florio Lucia, Silvia Spoto, Rosa Maria Chiantia, Alessandro Caserta, Raffaella Rosy Vescio, Flavio Davini, Lucrezia Bani, Elisabetta Riva, Massimo Ciccozzi, Silvia Angeletti

**Affiliations:** 1 Operative Research Unit of Laboratory, Fondazione Policlinico Universitario Campus Bio-Medico, Via Alvaro del Portillo, 200, 00128 Rome, Italy; m.ristori@policlinicocampus.it (M.V.R.); l.deflorio@policlinicocampus.it (D.F.L.); rosamaria.chiantia@unicampus.it (R.M.C.); a.caserta@unicampus.it (A.C.); raffaella.vescio@unicampus.it (R.R.V.); flavio.davini@unicampus.it (F.D.); lucreziabani3@gmail.com (L.B.); e.riva@policlinicocampus.it (E.R.); m.ciccozzi@unicampus.it (M.C.); 2 Department of Biomedical Sciences, University of Sassari, Viale San Pietro 43b, 07100 Sassari, Italy; fscarpa@uniss.it (F.S.); darsanna@uniss.it (D.S.); 3 Department of Veterinary Medicine, University of Sassari, 07100 Sassari, Italy; marcasu@uniss.it; 4 Infection Prevention Control and Infectious Disease Service, Fondazione Policlinico Universitario Campus Bio-Medico, 00128 Rome, Italy; n.petrosillo@policlinicocampus.it; 5 Fondazione Policlinico Universitario Campus Bio-Medico, Via Alvaro del Portillo, 200, 00128 Rome, Italy; g.longo@policlinicocampus.it; 6 Research Unit of Orthopaedic and Trauma Surgery, Department of Medicine and Surgery, University Campus Bio-Medico of Rome, Via Alvaro del Portillo, 21, 00128 Roma, Italy; 7 Diagnostic and Therapeutic Medicine Department, Fondazione Policlinico Universitario Campus Bio-Medico, Via Alvaro del Portillo, 200, 00128 Roma, Italy; s.spoto@policlinicocampus.it; 8 Unit of Virology, University Campus Bio-Medico of Rome, Via Alvaro del Portillo, 21, 00128 Rome, Italy; 9 Unit of Medical Statistic and Molecular Epidemiology, University Campus Bio-Medico of Rome, Via Alvaro del Portillo, 21, 00128 Rome, Italy; 10 Research Unit of Clinical Laboratory Science, Department of Medicine and Surgery, University Campus Bio-Medico of Rome, Via Alvaro del Portillo, 21, 00128 Rome, Italy

**Keywords:** *Klebsiella pneumoniae*, MDR, phylogenetic analysis, epidemiology investigation

## Abstract

Multidrug-resistant *Klebsiella pneumoniae* is a significant healthcare challenge that particularly affects vulnerable patients through opportunistic nosocomial infections. Surveillance is crucial for monitoring the prevalence of these infections. Eighty-four KPC *K. pneumoniae* strains (2019–2022) were collected from patients admitted in Fondazione Policlinico Universitario Campus Bio-Medico. Strains were identified by MALDI-TOF and tested for antimicrobial susceptibility, and gene amplification was performed to identify the different blaKPC variants. Phylogenetic reconstructions were carried out using Bayesian methods. Additionally, to create a Bayesian skyline plot (BSP), additional analyses were conducted, running a simulation of 100 million generations under a Bayesian skyline model along with the uncorrelated log-normal relaxed clock model. To identify potential subgroups within genetic clusters and evaluate genetic variability among sequences, principal coordinate analysis (PCoA) was performed. In total, 84 *Klebsiella pneumoniae* isolates were classified as multidrug-resistant (MDR), characterized by resistance to three or more antibiotic classes, including carbapenems, and testing positive for KPC gene presence, and were included in the study. The Bayesian evolutionary tree for *K. pneumoniae* showed strongly supported branches but no genetic structure related to sampling dates or hospital departments. Phylogenetic analysis revealing a 73-year evolutionary span of *K. pneumoniae* strains. PCoA analysis identified three genetic outliers from 2022 and one from 2021, indicating higher genetic distances. The Bayesian skyline plot revealed increased genetic variability peaking at the end of 2019, followed by stabilization from early 2020 onward, with no significant changes in genetic variability thereafter. Overall, the study found no genetic structure correlating with sampling date or hospital department, suggesting significant variability in pathogen introduction during the pandemic. The increase in multidrug-resistant *K. pneumoniae* was linked to the influx of severe COVID-19 cases, prolonged hospitalizations, and heightened broad-spectrum antibiotic use, which likely facilitated resistance development and transmission amidst altered infection control practices.

## 1. Introduction

Antibacterial resistance represents a significant public health threat in the European region, with over 670,000 infections and approximately 33,000 deaths reported annually in the EU/EEA due to this issue. The financial burden on healthcare systems is substantial, costing an estimated EUR 1.1 billion each year [1]. Regarding this problem, the COVID-19 pandemic has intensified the challenge of antimicrobial resistance (AMR), which was already a major global public health concern. Changes in antimicrobial usage, infection prevention, and health systems due to COVID-19 may influence the emergence and spread of AMR pathogens [2]. The Organisation for Economic Co-operation and Development (OECD) in fact forecasts a 72% increase in resistance to second-line antibiotics by 2030 compared to 2005 levels [3]. Furthermore, resistance rates vary across Europe, with southern and eastern regions showing the highest levels [3]. There is indeed growing concern about resistance to third-generation cephalosporins and carbapenems in *Klebsiella pneumoniae*, as well as carbapenem-resistant *Acinetobacter baumannii* (CR-Ab) and *Pseudomonas aeruginosa* (CR-Pa), in several European countries [3]. On this topic, a retrospective analysis by Cogliati Dezza et al. found that non-COVID-19 patients had a higher incidence of bloodstream infections caused by multidrug-resistant Gram-negative bacteria, particularly *Klebsiella pneumoniae*, whereas COVID-19 patients were more frequently infected with Acinetobacter baumannii [4]. Furthermore, Falcone et al. examined hypervirulent *Klebsiella pneumoniae* infections in COVID-19 patients, revealing a high mortality rate of 48.3% [5]. Many of these patients had strains that were resistant to certain antibiotics but remained susceptible to meropenem, underscoring the significant challenges posed by these infections during the pandemic [5]. Healthcare-associated infections (HAIs), commonly referred to as nosocomial infections, are widespread in hospitals and other medical facilities, posing serious risks to patient safety and contributing to a significant increase in morbidity and mortality. Due to its ability to cause severe nosocomial infections, multidrug-resistant (MDR) *Klebsiella pneumoniae* represents a significant challenge in healthcare settings. This pathogen has developed resistance to multiple classes of antibiotics, complicating both treatment and infection control efforts. In many cases, *Klebsiella pneumoniae* is responsible for these infections, taking advantage of patients’ weakened immune systems and pre-existing health conditions [6,7]. In fact, multidrug-resistant (MDR) strains of *Klebsiella pneumoniae* carry resistance genes that diminish the effectiveness of commonly used antibiotics. This resistance is favored by several factors, including the overuse and misuse of antibiotics, poor infection control practices, and genetic mutations. *K. pneumoniae*’s ability to rapidly acquire and transfer these resistance genes further exacerbates the problem. Infections caused by MDR strains are especially dangerous in critically ill patients or those undergoing invasive procedures, with the most common outcomes being pneumonia, urinary tract infections, and bloodstream infections. *K. pneumoniae* exhibits resistance through various mechanisms, including the production of extended-spectrum beta-lactamases (ESBLs) and carbapenemases like NDM and KPC [8]. With respect to that, a study found that 64.7% of *Klebsiella pneumoniae* strains were resistant to carbapenems, with significant rates of resistance to other antibiotics like piperacillin-tazobactam (95.5%) [9]. Precisely because of their resistance, genetic analysis reveals high variability among MDR strains, with unique genotypes contributing to their adaptability and virulence [9,10]. In this context, mobile genetic elements facilitate the spread of resistance genes, complicating treatment options [10].

Considering all the points discussed earlier, the rising threat of multidrug-resistant (MDR) *K. pneumoniae* and nosocomial infections implies the need for stringent infection control measures within healthcare facilities. Key strategies include rigorous hand hygiene, thorough cleaning of medical equipment and contaminated surfaces, and adherence to antibiotic stewardship programs, which are essential for preventing the transmission of MDR pathogens, as healthcare workers are often vectors for infection spread [11,12]. Regarding this, studies indicate that hospital plumbing and shared facilities can be reservoirs for MDR bacteria, necessitating focused cleaning efforts [13]. It is evident that, in light of what has been said, promoting responsible antibiotic use is crucial to mitigating resistance development and is a responsibility of the entire scientific community [14]. For monitoring nosocomial infections and the prevalence of MDR *K. pneumoniae*, comprehensive surveillance that allows targeted interventions in high-risk areas is essential [6,7,15]. Furthermore, studies and longitudinal analyses indicate that *K. pneumoniae* exhibits clonal spread within healthcare settings, particularly in residential aged care facilities, suggesting hospital-associated transmission dynamics [16]. The identification of high-risk clones, such as ST147, emphasizes the need for targeted interventions in specific healthcare environments [17]. Once again, infection prevention measures, including enhanced surveillance and control protocols, are crucial to reduce healthcare-associated infections [18,19,20]; but there is more to it. The emergence of hypervirulent strains complicates treatment and control efforts, necessitating continuous adaptation of strategies to combat this evolving threat [21,22].

Phylogenetic analysis serves as a valuable tool in tracing evolutionary relationships among organisms by comparing genetic sequences, typically of core genes or entire genomes [19]. In the context of MDR *K. pneumoniae*, phylogenetic analysis can reveal transmission dynamics, identify clonal lineages, and track the spread of resistance genes [19,20]. Numerous studies have provided compelling evidence for the necessity of systematic monitoring of MDR *K. pneumoniae* infections in healthcare environments. These investigations have underscored the significant burden of MDR infections, their association with healthcare-related risk factors, and the potential for rapid dissemination both locally and globally [23,24,25]. Molecular typing techniques, including pulsed-field gel electrophoresis and genome sequencing, are essential for tracking multidrug-resistant (MDR) *Klebsiella pneumoniae* infections [26]. These methods elucidate transmission dynamics, identify high-risk areas, and guide targeted interventions. Studies employing BOX-PCR and RAPD-PCR have demonstrated significant genetic variability among *K. pneumoniae* isolates, revealing unique genotypes and strong clonality, which aids in understanding transmission patterns in hospital settings [9,27,28,29].

The identification of specific sequence types (STs) associated with outbreaks, such as ST11 and ST258, enables precise targeting of high-risk areas and the development of tailored infection control strategies [29]. While these molecular techniques provide critical insights into *K. pneumoniae* transmission and resistance, challenges remain in their implementation in resource-limited settings, where access to advanced molecular tools may be restricted.

This article aims to highlight the vital importance of monitoring infections caused by multidrug-resistant (MDR) *Klebsiella pneumoniae* and stresses the need for comprehensive epidemiological surveillance to limit its spread. This study examines three key periods—pre-pandemic, pandemic, and post-pandemic—focusing on isolates collected in 2019, 2020, 2021, and 2022. Gaining insight into the dynamics and effects of MDR *K. pneumoniae* within the context of the COVID-19 pandemic is crucial for shaping effective infection control strategies moving forward.

## 2. Materials and Methods

### 2.1. Bacterial Isolates

Eighty-four *Klebsiella pneumoniae* strains producing KPC (*Klebsiella pneumoniae* carbapenemase) were isolated from monomicrobial infections between 2019 and 2022 in patients admitted to various wards at the Fondazione Policlinico Universitario Campus Bio-Medico in Rome, Italy. The strains were selectively isolated from colonies grown on McConkey agar and identified using MALDI-TOF (Bruker Daltonics GmbH, Bremen, Germany). Antimicrobial susceptibility testing was conducted as previously described [30], by semi-automated broth dilution method. For each isolate, details were recorded on the source of isolation, the hospital area (including both the ward and the patient’s specific bed), the year of isolation, and the antibiotic resistance patterns, as summarized in Table 1. The study was conducted according to the guidelines of the Declaration of Helsinki and approved by the Ethical Committee of the Fondazione Policlinico Universitario Campus Bio-Medico of Rome (28.16 TS Com Et CBM). Informed consent was not required due to the retrospective design of the study.

### 2.2. blaKPC Gene Sequencing

The gene was amplified using the forward primer KPC-F107 with the reverse primer KPC-R860, by polymerase chain reaction (PCR), producing a product of 753 bp, and sequencing [31] was performed to identify the different blaKPC variants, as previously described [30].

### 2.3. Phylodynamics

To investigate the evolutionary patterns of *Klebsiella pneumoniae* within and among different hospital departments throughout the COVID-19 pandemic period, the dataset (n = 84) includes sequences isolated across four different years (2019–2022) from all available hospital departments.

The process involved aligning the sequences using the L-INS-I algorithm implemented in the software Mafft 7.471 [32] and further refining them manually with Unipro UGENE v.35 [33]. The most suitable model for genome evolution was determined using jModeltest 2.1.1 [33,34], and the evolutionary relationships were examined using MrBayes 3.2.7 [35].

Two independent runs were executed, each comprising four Metropolis-coupled Markov-chain Monte Carlo (MCMCMC) simulations, including one cold chain and three heated chains. These runs were concurrently conducted for 5,000,000 generations, with tree samples taken every 1000 generations, according to Scarpa et al. [36]. The initial 25% of the 10,000 trees sampled were eliminated as burn-in. Nodes that displayed a posterior probability exceeding 0.95 were deemed statistically significant. The resulting tree illustrating the evolutionary connections was displayed using FigTree 1.4.0 [37]. To reconstruct the time-calibrated tree and to estimate divergence times among variants, Bayesian Inference (BI) was also employed using the software program BEAST 1.10.4 (Drummond and Rambaut, 2007) [38]. This method, grounded in the neutral theory of molecular evolution, allowed for minimum age molecular dating, where branch lengths corresponded to elapsed time. The analysis comprised runs of 200 million generations, exploring various demographic and clock models, with an ESS ≥ 200 considered in the screening process. To select the best demographic model, the Bayes Factor test [39] was performed as described in Mugosa et al. [40]. The resulting maximum clade credibility tree was generated and edited using TreeAnnotator (BEAST package) and FigTree 1.4.0 [37], respectively. Additionally, to create the Bayesian skyline plot (BSP), additional analyses were conducted, running a simulation of 100 million generations under the Bayesian skyline model along with the uncorrelated log-normal relaxed clock model.

To identify potential subgroups within genetic clusters and evaluate genetic variability among sequences, principal coordinate analysis (PCoA) using GenAlEx 6.5 [41] was conducted. The analysis was performed on a pairwise genetic matrix built on p-distances. This analysis aimed to assess dissimilarities in genetic variability among the sequences studied and allow identification of genetic structures based on the nucleotide composition.

## 3. Results

### 3.1. Isolates

All 84 isolates were classified as MDR (strains were resistant to carbapenems, cephalosporin, fluoroquinolones, and penicillin), indicating resistance to three or more antibiotic classes, including carbapenems, and tested positive for KPC.

The strains were isolated from various biological samples form patients admitted to different wards of the hospital as reported in Table 1. Specifically, in the years 2020 and 2021, some strains were isolated from patients with concurrent SARS-CoV-2 infection who were subsequently admitted to the COVID-19 wards of the hospital.

In 22 out of 84 patients (26%), MDR *K. pneumoniae* and KPC were the cause of disseminated infection and were isolated from blood cultures, and 2 out of 84 (2.4%) patients exhibited catheter-related bloodstream infections (CRBSI). In 28 out of 84 cases (33%) this pathogen was the cause of urinary tract infections, while in 9 out of 84 cases it was the cause of surgical infections, in 5 out of 84 it caused cutaneous necrotic infections (ulcers) in patients admitted in medical wards, in 12 out of 84 it was the cause of respiratory tract infections, and in 6 out of 84 cases MDR *K. pneumoniae* were isolated in drainage or biological fluid (ascites) (Table 1). The most common wards for the isolation of *K. pneumoniae* resistant strains were medical wards, with 41 out of 84 cases (49%), surgical wards, with 17 out of 84 cases (20%),and the intensive care unit, with 18 out of 84 cases (21.5%). In 8 out of 84 cases (9.5%), isolates were from patients admitted to COVID-19 wards, including those receiving intensive and semi-intensive care (Table 1).

### 3.2. Phylodynamics and Phylogenetic Reconstruction of KPC Klebsiella pneumoniae Strain Time-Scaled Maximum Clade Credibility Phylogenetic Tree

**Figure 1:**
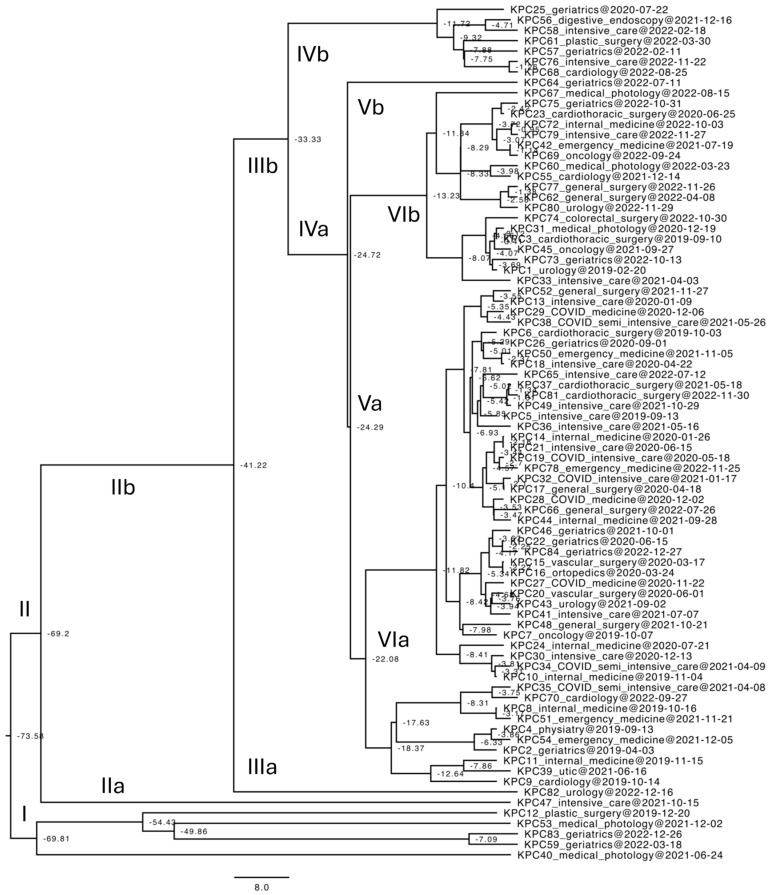
Phylogenetic reconstruction of KPC *Klebsiella pneumoniae* strains. Time-scaled maximum clade credibility phylogenetic tree of n = 82 sequences. Node values are expressed in years and represent the divergence time of the related clade. All nodes are considered highly supported presenting values of posterior probabilities upper to 0.9.

The Bayes factor test demonstrated that the coalescent Bayesian skyline, under the lognormal uncorrelated relaxed clock model, exhibited notably superior conformity to the data in contrast to other models tested (2lnBF = 11.2). Both the phylogenetic tree derived from MrBayes and the time-scaled phylogenetic tree generated using the software program BEAST exhibit identical topologies. Hence, only the latter is presented here (Figure 1). The Bayesian evolutionary tree, constructed from a dataset encompassing all available *Klebsiella pneumoniae* (Figure 1), exhibits strongly supported branches (posterior probabilities 0.90 ≤ 1). As represented in Figure 1, the following data can be inferred. The origin of the tree is dated to −73.58 years, meaning more than 73 years ago. This implies that the most recently isolated strains, located at the terminals of the tree, have undergone evolution in terms of accumulated mutations equivalent to approximately 73 years compared to their ancestor. From the origin, two clusters are distinguished, dated respectively to −69.81 and −69.2 years ago, thus more or less contemporaneous, consisting of 5 isolates (Clade I) and 79 isolates (Clade II). Clade I includes KPC-12, KPC-53, KPC-83, KPC-59, and KPC-40, forming a separate group. It can be observed that, in December 2019, strain KPC-12 was isolated from the plastic surgery department, and from the tree, it is evident that its circulation extended in December 2021 to the medical pathology department, where strain KPC-53 was isolated. In March 2022, the circulation of these strains continued in the geriatrics department with the KPC-59 isolate, persisting throughout 2022, with the KPC-83 strain isolated in December 2022. Clade II is divided into two sub-branches (IIa and IIb), consisting of 1 isolate (KPC-47) and 78 isolates, respectively. The strain from branch IIa was isolated in the ICU in October 2021 and is entirely separate from the other strains in clade II, suggesting it did not undergo clonal transmission but rather was limited to the single patient in whom it was identified. Branch IIb divides into two additional sub-branches (IIIa and IIIb). Branch IIIa consists of a single strain, KPC-82, isolated in December 2022 in the urology department, which, again, likely did not undergo clonal transmission but was limited to the patient in whom it was identified. On the other hand, branch IIIb includes numerous strains that further divide into two sub-branches, IVa (71 isolates) and IVb (7 isolates). In branch IVb, strain KPC-25, isolated in July 2020 from the geriatrics department, stands out, as it is separate from the others, indicating no subsequent clonal expansion. The remaining six strains of branch IVb include KPC-56, KPC-58, KPC-61, KPC-57, KPC-76, and KPC-68. Here, a close correlation is observed, both in terms of tree position and evolutionary timeline. In particular, all were isolated between the end of 2021 and 2022, and their presence in the hospital was observed within a narrow timeframe, supporting their clonality. Furthermore, as previously reported for Clade I, circulation is confirmed within the geriatrics, ICU, and plastic surgery departments, with the cardiology and digestive endoscopy departments also involved. Specifically, it is observed that strain KPC-56, first isolated in December 2021 from the digestive endoscopy department, is closely related to strain KPC-58, isolated from the ICU in February 2022. Subsequently, KPC-57 and KPC-61, closely related, were isolated from the geriatrics and plastic surgery departments, respectively, in February and March 2022. Lastly, KPC-68 and KPC-76 were isolated from the cardiology and ICU departments in August and November 2022, respectively. Most of the isolates circulating in the hospital from 2020 to 2022 are represented in branch IVb. If we go further in the analysis, we can distinguish branch Vb, corresponding to strain KPC-64, which is located outside of branch Va, which includes all the other isolates, further dividing into branches VIa and VIb, where the isolates do not follow any particular circulation pattern but are intermixed both in terms of timeline (year of isolation) and space (department of isolation). The only observation is that the strains circulating in the COVID-19 wards are all located in branch VIa, confirming that they circulated during a similar period (the pandemic years) and are temporally older than the strains of branch VIb: in fact, node VIa is dated to −22 years versus −13 years for node VIb. From an evolutionary point of view, there is a gap of about 30 years between Clade II and branch IIb (69 vs. 41 years). There is a gap of about 9 years between the nodes of branches IV and V (−33.3 vs. −24.3 years), while there is a smaller time difference between node V and node VI. Specifically, Va and VIa are almost contemporaneous, dated to −24 years vs. −22 years, while there is a longer gap between Va and VIb, of approximately 9 years (−24 years vs. −13 years). The estimated evolutionary rate amounts to 4.1 × 10^−3^ substitutions/site/year, with a 95% HPD (high posterior density) of 5.8 × 10^−4^–7.1 × 10^−3^.

### 3.3. Principal Coordinate Analysis (PCoA)

**Figure 2:**
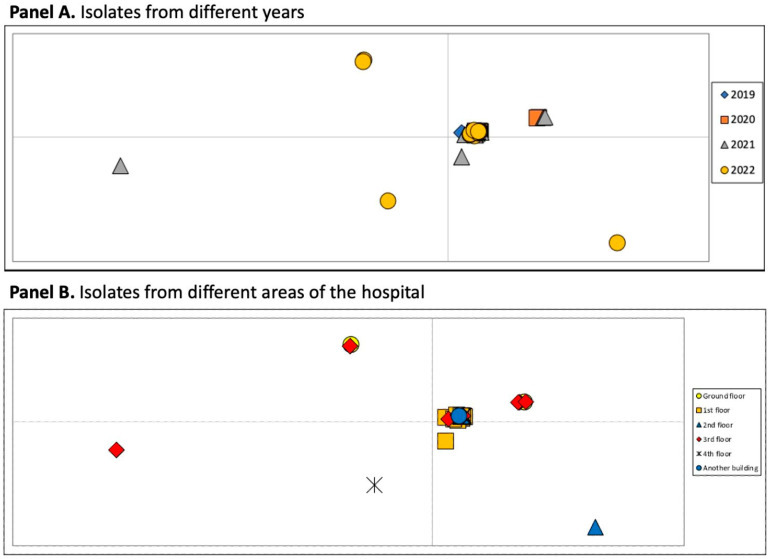
Principal coordinate analysis of KPC *Klebsiella pneumoniae* strains. The cumulative variability explained by the first three axes amounts to 89.81% (axis 1: 60.42; axis 2: 21.61; axis 3: 7.78). (**A**) The groups were set a priori in accordance with the sampling years (2019 12 samples, 2020 19 samples, 2021 25 samples, and 2022 28 samples). (**B**) The groups were set a priori in accordance with the location of the department from which they were isolated (ground floor 40 samples, first floor 12 samples, second floor 5 samples, third floor 21 samples, fourth floor 4 samples, and another building 1 sample).

From PCOA analysis, the following observation can be inferred, as evidenced in Figure 2. The PCoA by year (panel A) generally highlights a lack of structured circulation, as most of the isolates from different years are positioned in the central area of the plot. However, the graph shows that some strains with more recent isolation dates, particularly from late 2021 and 2022, are positioned differently and further away from the central group. As for the PCoA analysis shown in panel B, there is a complete lack of structured circulation, as the isolates are almost exclusively located in a single point on the graph, except for a few sporadic cases.

### 3.4. Bayesian Skyline Plot (BSP)

**Figure 3:**
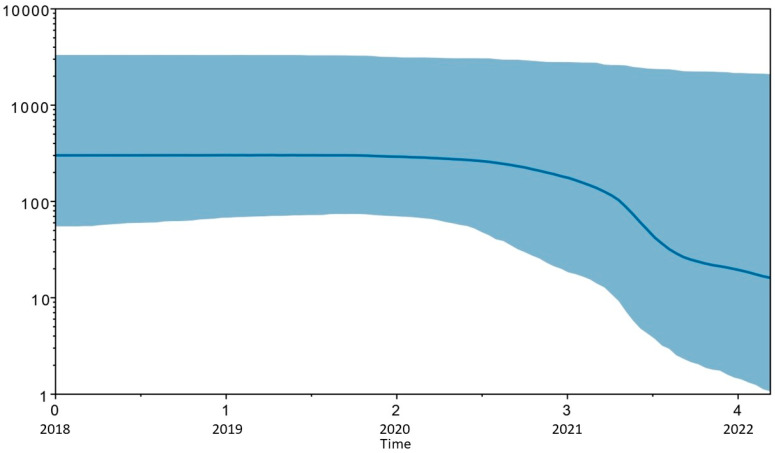
Bayesian skyline plot of KPC *Klebsiella pneumoniae* strains. The genetic variability and viral effective size (y-axis) are shown as a function of time (x-axis). The solid area represents the 95% high posterior density (HPD) region. Values of time are expressed in years before the most recent sample (i.e., 2022).

By this analysis, the curve schematized in Figure 3 was obtained, and the following data were inferred. An exponential increase in the number of isolates during the first years of sampling (2019–2020), followed by the reaching a plateau in the subsequent years (2021–2022), was evidenced.

## 4. Discussion

Epidemiological investigation plays a crucial role in understanding and combating antibiotic resistance, a growing global health threat. These investigations help identify high-risk areas or populations where resistant strains are prevalent, allowing for targeted interventions that minimize further transmission. Moreover, epidemiological data on resistance trends provide insights into the effectiveness of current antibiotic policies and enable health authorities to adjust guidelines to better manage resistant infections. Through continuous monitoring, surveillance, and data collection, epidemiological research supports the development of predictive models that can anticipate future resistance trends. This proactive approach is essential for guiding pharmaceutical research into new antibiotics and alternative treatments, as well as for informing public health campaigns that raise awareness about the responsible use of antibiotics. Combining classical epidemiological investigations with molecular analysis, particularly through phylogenetic analysis, is crucial for gaining a deeper understanding of the circulation of multidrug-resistant (MDR) bacteria. The use of phylogenetic analysis by examining the genetic sequences of bacterial isolates in healthcare settings to track the circulation of multidrug-resistant (MDR) bacteria represents a powerful complement to traditional epidemiological methods [42]. In hospital environments, where the spread of resistant bacteria poses significant challenges to patient safety and infection control, phylogenetic analysis offers a more detailed and precise understanding of how these pathogens evolve and transmit. This approach can detect subtle genetic changes that may go unnoticed with traditional methods, enabling the identification of hidden transmission chains or clonal expansions of specific resistant strains.

While classical epidemiology provides valuable insights into infection patterns, risk factors, and transmission dynamics, it can sometimes lack the depth needed to uncover the full scope of bacterial spread and mutation [20,30,43,44,45]. During the COVID-19 pandemic, a variety of infection prevention and control (IPC) measures was implemented to reduce nosocomial transmission of the virus among patients and healthcare workers (HCWs) [46]. Comparative studies of pre-pandemic and pandemic periods have revealed an increase in certain healthcare-associated infections (HAIs) at specific facilities, often linked to the widespread empirical use of broad-spectrum antibiotics in COVID-19 patients [47]. However, the strict enforcement of IPC protocols to prevent the spread of COVID-19 has also contributed to a reduction in some bacterial HAIs in certain settings. Preliminary research suggests that, although COVID-19 patients are at a relatively low yet significant risk of developing HAIs after hospital admission, this risk escalates with disease severity and prolonged hospitalization. Additionally, some of the bacterial HAIs identified in these patients have demonstrated antibiotic resistance, including multidrug resistance. Trends in infection rates from before and during the pandemic indicate a rise in certain HAIs across various healthcare settings, with the extensive use of broad-spectrum antibiotics being a key contributing factor [48].

During the first 18 months of the COVID-19 pandemic, microbiologically confirmed antimicrobial resistance (AMR) was markedly higher among patients with bacterial co-infections. The most prevalent resistant pathogens included carbapenem-resistant *Acinetobacter baumannii* (CRAB), methicillin-resistant *Staphylococcus aureus* (MRSA), *Klebsiella pneumoniae*, and *Pseudomonas aeruginosa*. However, it is important to recognize that scaling up and intensifying infection prevention and control (IPC) protocols is not always feasible. For instance, isolating all symptomatic patients in specialized wards for infectious diseases may not be practical, given resource constraints. Furthermore, healthcare workers face increased risk of burnout, particularly when the patient-to-nurse ratio surpasses 2:1, a scenario exacerbated by excessive workloads. While enhanced IPC measures are critical for managing healthcare-associated infections (HAIs), a significant challenge lies in balancing these protocols with other pressing clinical demands. There is an interface between COVID-19 and bacterial healthcare-associated infections [49]. Collaboration among healthcare providers, researchers, and policymakers is vital to combat the spread of MDR *Klebsiella pneumoniae* and mitigate the impact on public health.

Our study provides strong evidence of bacterial evolution and transmission through phylogenetic analysis of KPC-producing *Klebsiella pneumoniae* strains, using a time-scaled maximum clade credibility tree to infer their evolutionary origins and transmission patterns. The phylogeny suggests that the lineage originated over 73 years ago, exhibiting significant genetic divergence among the isolated strains. The analysis identified two main clusters, Clade I and Clade II, with distinct transmission timelines across different hospital departments. The rapid evolutionary changes observed are likely a result of selective pressures, such as the misuse of antibiotics, particularly during the COVID-19 pandemic, which accelerated bacterial evolution.

Interestingly, the evolutionary timeline is relatively short—under 10 years—implying that a strong selective force, likely the extensive use of antibiotics during the pandemic (2020–2022), acted as a catalyst for rapid adaptation. Our findings indicate that most strains circulating between 2020 and 2022 fall within branch IVb of the phylogenetic tree, while isolates from the COVID-19 wards are clustered in branch VIa, suggesting a temporal link to the pandemic years. These isolates are evolutionarily older than those in branch VIb, as evidenced by node VIa being dated to approximately 22 years ago, compared to 13 years for node VIb.

From an evolutionary perspective, a significant temporal gap of about 30 years exists between Clade II and branch IIb (69 vs. 41 years), a disparity that gradually narrows, reflecting accelerated evolutionary rates in response to nosocomial selective pressures. For instance, the time gap between branches IIb and IIIb is approximately 8 years (−41 vs. −33 years), with a similar interval observed between IIIb and IVa (−33 vs. −24 years). Branch IVa shows more recent evolutionary developments, as most of its isolates date to 2022, with the exception of KPC-25, an outlier from 2020. Additionally, there is a temporal gap of about 9 years between branches IV and V (−33.3 vs. −24.3 years), while the interval between nodes V and VI is shorter, indicating nearly contemporaneous emergence for Va and VIa (−24 vs. −22 years), whereas Va and VIb are separated by 9 years (−24 vs. −13 years).

This evolutionary trajectory reveals that branch VIa comprises older strains, including those circulating in the COVID-19 wards, while branch VIb is dominated by more recent isolates, with 11 of 18 strains dated to 2022. In contrast, only 6 of the 48 isolates in VIa were identified in 2022. Despite the stringent isolation and sanitation measures implemented during the pandemic, the strains from COVID-19 wards did not remain confined to these areas. This suggests that the non-structured dissemination of strains, both across departments and over time, was primarily driven by the selective pressure exerted by widespread antibiotic use between 2019 and 2022, overriding the containment efforts in place during the pandemic [6].

Further principal coordinate analysis (PCOA) revealed that isolates from late 2021 and 2022 diverged significantly from older strains, suggesting evolutionary adaptation in response to enhanced containment measures. This divergence is likely a consequence of more rigorous surveillance of resistant strains and more judicious use of antibiotics. The data indicate that these more recent isolates have undergone distinct evolutionary changes, a pattern consistent with the containment strategies implemented after 2020. This observation aligns with the phylogenetic tree, where isolates from different time periods and hospital departments show unstructured circulation, with strains intermingling irrespective of their isolation date or location. Notably, strains from 2022 exhibit accelerated evolutionary rates and are frequently concentrated in a specific branch of the tree (VIb).

These findings were further corroborated by Bayesian skyline plot (BSP) analysis, which demonstrated an exponential rise in the number of isolates between 2019 and 2020, followed by a plateau in 2021–2022. This suggests a stabilization in the circulation of KPC-producing strains within the hospital setting. Despite the implementation of COVID-19 containment measures, including patient isolation, the pandemic period saw a significant increase in the spread of multidrug-resistant (MDR) and KPC strains, likely driven by widespread inappropriate antibiotic use, as reported in other scientific studies [50]. This underscores a critical issue observed during the COVID-19 pandemic, where the spread of multidrug-resistant (MDR) and KPC strains significantly increased. As reported by several other authors, this surge can be attributed to the widespread and often inappropriate use of antibiotics, a consequence of the heightened pressure on healthcare systems during the pandemic. Studies from various sources have highlighted how the overprescription or incorrect use of antibiotics, particularly in treating viral infections like COVID-19, contributed to the development and spread of resistant bacterial strains. This phenomenon not only complicates treatment of infections but also emphasizes the urgent need for more stringent antibiotic stewardship practices to curb further resistance and protect public health [51,52,53]. In the subsequent years, with the stabilization of the pandemic and the return to more responsible antibiotic use through strengthened antibiotic stewardship protocols, the exponential growth of resistant isolates was halted, leading to the observed plateau in their circulation. Rapid microbiological diagnostics have a significant impact on the appropriate use of antibiotics and antimicrobial stewardship. By enabling quicker and more accurate identification of pathogens, these diagnostic tools help clinicians distinguish between bacterial and viral infections, preventing unnecessary antibiotic prescriptions for viral illnesses. Moreover, rapid diagnostics can guide more targeted antibiotic therapies, reducing the use of broad-spectrum antibiotics and minimizing the risk of antibiotic resistance. In turn, this leads to improved patient outcomes, shorter hospital stays, and a reduction in healthcare costs, highlighting the critical role of rapid diagnostics in promoting responsible antibiotic use [54,55,56].

## 5. Conclusions

The use of phylogenetic analysis in healthcare settings to track the circulation of multidrug-resistant (MDR) bacteria represents a powerful complement to traditional epidemiological methods. In hospital environments, where the spread of resistant bacteria poses significant challenges to patient safety and infection control, phylogenetic analysis offers a more detailed and precise understanding of how these pathogens evolve and spread.

When combined with classical epidemiological data, phylogenetics enhances microbiological surveillance and supports the implementation of more effective prevention and control measures. Ultimately, the integration of molecular and epidemiological techniques strengthens the overall response to antimicrobial resistance in healthcare settings, improving both patient outcomes and infection control strategies.

## Figures and Tables

**Table 1:** Isolate descriptions: site of isolation, hospital ward, year of isolation, pattern of antibiotic resistance, and hospital area where the patients were admitted.

Site of Infection	Ward	Year	Resistance	Hospital Area and Bed
syringe aspirate	Urology	2019	MDR, KPC	IIE
hepatic drainage	Geriatrics	2019	MDR, KPC	IIIO
surgical wound	Cardiothoracic Surgery	2019	MDR, KPC	IO
urine culture	Physiatry	2019	MDR, KPC	CESA
CVC	Intensive Care Unit	2019	MDR, KPC	TI
pleural fluid	Cardiothoracic Surgery	2019	MDR, KPC	IO
urine culture	Oncology	2019	MDR, KPC	IE
syringe aspirate	Internal Medicine	2019	MDR, KPC	IIIE
urine culture	Cardiology	2019	MDR, KPC	TI
urine culture	Internal Medicine	2019	MDR, KPC	IIIO
urine culture	Internal Medicine	2019	MDR, KPC	IIIO
syringe aspirate	Intensive Care Unit	2019	MDR, KPC	TI
tracheoaspirate	Intensive Care Unit	2020	MDR, KPC	TI
urine	Internal Medicine	2020	MDR, KPC	IO
necrotic tissue	Vascular Surgery	2020	MDR, KPC	IIIO
surgical wound	Orthopedics	2020	MDR, KPC	IVO
urine	General Surgery	2020	MDR, KPC	IVO
urine	Intensive Care Unit	2020	MDR, KPC	TI
blood culture	COVID-19 Intensive Care Unit	2020	MDR, KPC	TI
blood culture	Vascular Surgery	2020	MDR, KPC	IIIO
ulcer	Intensive Care Unit	2020	MDR, KPC	TI
surgical wound	Geriatrics	2020	MDR, KPC	IO
tissue	Cardiothoracic Surgery	2020	MDR, KPC	IO
urine	Internal Medicine	2020	MDR, KPC	IIIE
tracheobronchial aspirate	Geriatrics	2020	MDR, KPC	IO
urine	Geriatrics	2020	MDR, KPC	P0
tracheobronchial aspirate	COVID-19 Ward	2020	MDR, KPC	P0
urine	COVID-19 Ward	2020	MDR, KPC	P0
urine	COVID-19 Ward	2020	MDR, KPC	P0
urine	Intensive Care Unit	2020	MDR, KPC	TI COVID-19
urine	Medical Pathology Unit	2020	MDR, KPC	IIIO
tracheoaspirate	COVID-19 Intensive Care Unit	2021	MDR, KPC	TI COVID-19
tracheobronchial aspirate	Intensive Care Unit	2021	MDR, KPC	TI COVID-19
urine culture	COVID-19 Semi-Intensive Care Unit	2021	MDR, KPC	SI COVID-19
urine culture	COVID-19 Semi-Intensive Care Unit	2021	MDR, KPC	SI COVID-19
urine culture	Intensive Care Unit	2021	MDR, KPC	TI COVID-19
wound	Cardiothoracic Surgery	2021	MDR, KPC	IO
tracheobronchial aspirate	COVID-19 Semi-Intensive Care Unit	2021	MDR, KPC	SI COVID-19
urine culture	Coronary Intensive Care Unit UTIC	2021	MDR, KPC	UTIC
urine culture	Medical Pathology Unit	2021	MDR, KPC	IIIO
CVC	Intensive Care Unit	2021	MDR, KPC	TI
urine culture	Emergency Medicine	2021	MDR, KPC	P0
urine culture	Urology	2021	MDR, KPC	IIIO
blood culture	Internal Medicine	2021	MDR, KPC	P0
blood culture	Oncology	2021	MDR, KPC	IIIE
blood culture	Geriatrics	2021	MDR, KPC	P0
blood culture	Intensive Care Unit	2021	MDR, KPC	TI
wound	General Surgery	2021	MDR, KPC	IIE
ascitic fluid	Intensive Care Unit	2021	MDR, KPC	IIIO
blood culture	Emergency Medicine	2021	MDR, KPC	P0
blood culture	Emergency Medicine	2021	MDR, KPC	P0
blood culture	General Surgery	2021	MDR, KPC	IIIO
blood culture	Medical Pathology Unit	2021	MDR, KPC	IIIO
blood culture	Emergency Medicine	2021	MDR, KPC	P0
urine culture	Cardiology	2021	MDR, KPC	IE
blood culture	Digestive Endoscopy Unit	2021	MDR, KPC	P0
drainage fluid	Geriatrics	2022	MDR, KPC	P0
BAL	Intensive Care Unit	2022	MDR, KPC	TI
tracheobronchial aspirate	Geriatrics	2022	MDR, KPC	P0
urine culture	Medical Pathology Unit	2022	MDR, KPC	IIIO
surgical wound	Intensive Care Unit	2022	MDR, KPC	IIIO
drainage fluid	General Surgery	2022	MDR, KPC	IIE
bile	General Surgery	2022	MDR, KPC	IIE
blood culture	Geriatrics	2022	MDR, KPC	TI
BAL	Intensive Care Unit	2022	MDR, KPC	TI
blood culture	General Surgery	2022	MDR, KPC	IIE
blood culture	Medical Pathology Unit	2022	MDR, KPC	IIIO
blood culture	Cardiology	2022	MDR, KPC	IE
ascitic fluid	Oncology	2022	MDR, KPC	IIIE
urine culture	Cardiology	2022	MDR, KPC	IE
urine culture	Intensive Care Unit	2022	MDR, KPC	IIIO
blood culture	Internal Medicine	2022	MDR, KPC	P0
blood culture	Geriatrics	2022	MDR, KPC	P0
urine culture	General Surgery	2022	MDR, KPC	IIE
urine culture	Geriatrics	2022	MDR, KPC	P0
blood culture	Intensive Care Unit	2022	MDR, KPC	TI
surgical wound	General Surgery	2022	MDR, KPC	IIIO
sputum	Emergency Medicine	2022	MDR, KPC	P0
urine culture	Intensive Care Unit	2022	MDR, KPC	TI
blood culture	Urology	2022	MDR, KPC	IVO
surgical wound	Cardiothoracic Surgery	2022	MDR, KPC	IO
blood culture	Urology	2022	MDR, KPC	IVO
sputum	Geriatrics	2022	MDR, KPC	IIIO
blood culture	Geriatrics	2022	MDR, KPC	P0

CESA = separate building named the CESA building; TI = intensive care unit; IO = first floor west side; IIIO = third floor west side; IVO = fourth floor west side; IE = first floor east side; IIE = second floor east side; IIIE = third floor east side; TI COVID-19 = intensive care unit for COVID-19 patients; SI COVID-19 = semi-intensive care unit for COVID-19 patients; P0 = ground floor; UTIC = coronary intensive care unit.

## Data Availability

Data are unavailable due to privacy and ethical restrictions.
